# Do Older People Expect Reciprocity When Providing Care? Evidence From a National Cohort

**DOI:** 10.1093/geroni/igaa057.106

**Published:** 2020-12-16

**Authors:** Cheng Shi, Yajing Zhu, Gloria Hoi Yan Wong

**Affiliations:** 1 The University of Hong Kong, Hong Kong, China; 2 University of Cambridge, Cambridge, England, United Kingdom

## Abstract

Background: The debate on whether caregiving is selfless or self-serving is unresolved. From the perspective of caregiving as a moral responsibility, the motivation to provide care is altruistic. The exchange or reciprocity perspective suggests that care is provided with an expectation to be cared for in the future. The context of filial obligation and rapid socio-economic transformation in recent decades in urban and rural China provided a unique opportunity to investigate these two driving forces. Methods: We tested the effects of caregiving experience in older persons on their care expectation from their children using data from the China Longitudinal Aging Social Survey (n=8,308). Using a multiple-indicator multiple-cause model, a special form of structural equation modelling, we examined the effect of caring for one’s older parents, helping adult children with housework, and caring for grandchildren on older persons’ care expectation from their children. Results: Older persons in urban areas had higher expectation for future care from their children if they have helped them with housework or took care of their grandchildren, whereas their experience of taking care of their own parents is related to a lower care expectation from their children. Although a similar pattern was observed in rural areas, only the experience of helping adult children with housework significantly increased care expectation. Discussion: Both altruistic and exchange motivations are involved in caregiving. Caring for older parents was more likely altruistic. Caring for an adult child in both urban and rural areas, and grandparenting in urban areas, were motivated by reciprocity.

